# Identification and validation of bile exosomal microRNA signatures for diagnosing acute rejection in liver transplant recipients

**DOI:** 10.3389/fgene.2026.1877181

**Published:** 2026-06-29

**Authors:** Wenjing Wang, Hui Wang, Wen Li, Renchi Fu, Bo Wang, Bo Guo

**Affiliations:** 1 Surgical Intensive Care Unit, The First Affiliated Hospital of Xi’an Jiaotong University, Xi’an, China; 2 Department of Cell Biology and Genetics, School of Basic Medical Sciences, Xi’an Jiaotong University Health Science Center, Xi’an, China; 3 Department of Hepatobiliary Surgery, The First Affiliated Hospital of Xi’an Jiaotong University, Xi’an, China

**Keywords:** acute rejection, bile, exosomes, liver transplantation, miRNA

## Abstract

**Introduction:**

Acute rejection (AR) remains a major complication affecting graft function and recipient prognosis after liver transplantation (LT). Bile exosomal microRNAs (miRNAs) possess organ specificity, local enrichment, and high stability, making them promising non-invasive liquid biopsy biomarkers for the early detection of AR.

**Methods:**

In this study, bile samples were collected from 30 LT recipients with AR and 30 recipients with stable graft function (non-AR). Exosomes were isolated via ultracentrifugation and characterized. Total RNA was extracted and subjected to small RNA sequencing, followed by bioinformatics analysis. Candidate differentially expressed miRNAs were then validated using Real-Time Quantitative PCR (qRT-PCR) in an expanded cohort.

**Results:**

Sequencing revealed 63 significantly upregulated and 4 downregulated miRNAs in the AR group compared with the non-AR group. Target genes of candidate differentially expressed miRNAs were enriched in key immune-regulatory pathways, including PI3K-Akt and MAPK signaling. qRT-PCR validation further confirmed that the expression levels of miR-181a-5p, miR-200c-3p, and miR-192-5p in bile exosomes were significantly higher in the AR group (P < 0.0001).

**Discussion:**

Our findings identify and validate bile exosomal miRNA signatures for AR in LT recipients, supporting these miRNAs as a low-risk, organ-specific liquid biopsy strategy for AR diagnosis, with potential for clinical translation in post-transplant monitoring.

## 1 Introduction

Liver transplantation (LT) has become the only effective treatment for end-stage liver disease, and acute rejection (AR) is an important complication that affects the function of the transplanted liver and the prognosis of the recipient (Tang et al., 2024). The clinical manifestations of AR are atypical and easily confused with other diseases. The traditional indicators currently used in clinical practice have poor specificity and sensitivity. Pathological biopsy remains the gold standard for AR diagnosis, but it has disadvantages such as invasiveness, high false negatives, and multiple complications (Rastogi, 2022). When pathological abnormalities occur, the graft often has irreversible damage, leading to poor outcomes. Therefore, there is an urgent need to identify novel biomarkers for the early diagnosis of AR after LT, as this holds significant clinical value for improving outcomes.

Studies have shown that exosomes can serve as highly specific biomarkers in liquid biopsy (Yu et al., 2022). Exosomes are membrane vesicles with a diameter of 30–150 nm, containing stable biological information derived from their source cells, such as proteins, mRNAs, and microRNA (miRNAs), and play important roles in intercellular signal transduction (Gurung et al., 2021). miRNAs are non-coding, regulatory small RNAs that affect multiple processes including mRNA stability and the initiation and progression of protein translation, thereby exerting crucial regulatory functions in various physiological and pathological processes (Vishnoi and Rani, 2023).

Liquid biopsy, as an important branch of *in vitro* diagnosis, diagnoses and monitors tumors and other diseases by capturing and detecting biomarkers in various body fluids. Bile is generated by the liver, flows through the bile duct, contact with the bile duct epithelium directly, and contains abundant metabolites associated with the metabolic pathways of bile duct epithelial cells (Stieger, 2022). For hepatobiliary diseases, bile is a body fluid that is closer to the pathological source than blood and can contain higher levels of secreted or shed biomarkers (Gowda, 2010; Kuwatani and Sakamoto, 2022; Li et al., 2022; Alvaro, 2009; Farina et al., 2009; Liu et al., 2025). Bile samples can be obtained through various procedures such as intraoperative liver transplantation, postoperative T-tube, percutaneous biliary drainage, or retrograde cholangiopancreatography. Exosomal miRNAs are relatively stable in bile, and the concentration of relevant biomarkers in bile specimens is higher than in other body fluids (Groot and Lee, 2020; Olaizola et al., 2018). Moreover, bile-derived exosomal miRNAs directly reflect the local inflammatory and immune status of the graft, with minimal interference from distant organ lesions. Therefore, bile exosomal miRNAs hold potential diagnostic value for hepatobiliary diseases, particularly for benign and malignant biliary disorders (Shu et al., 2024; Fu et al., 2024). However, studies on the use of bile exosomal miRNAs as biomarkers to assist in the diagnosis of AR after LT remain scarce.

Based on this, this study aimed to elucidate the differential expression profile of miRNAs in bile exosomes from recipients with AR after LT compared to recipients with good postoperative recovery and stable liver function (non-AR), as well as the functions of target genes and the related signaling pathways. This provides an important basis for exploring the feasibility of using bile exosomal miRNAs as liquid biopsy markers for AR after LT.

## 2 Results

### 2.1 Clinical characteristics

To prevent AR, all recipients received a postoperative combined immunosuppressive regimen consisting of tacrolimus/cyclosporine, mycophenolate mofetil, and prednisone. The baseline characteristics of the liver transplant recipients were detailed in Table 1. The study found no significant differences between the AR and non-AR groups in terms of age, sex, body mass index, etiology, Child-Pugh score, MELD score (Model for End-Stage Liver Disease), cold/warm ischemia time, and blood type compatibility (P > 0.05).

**TABLE 1:** Clinical baseline characteristics of recipients.

Variables	AR N = 30	Non-AR N = 30	
Age (year)	49.56 ± 9.78	50.23 ± 9.08	
Sex (M/F)	23/7	24/6	
Body mass index (kg/m^2^)	24.67 ± 3.98	23.98 ± 4.79	
Etiology Cirrhosis Hepatitis B cirrhosis Hepatitis C cirrhosis Alcoholic cirrhosis Others Hepatocellular carcinoma Liver failure	23 13 4 2 4 5 2	21 14 4 1 2 6 3	
Child-plug score B C	21 9	22 8	
MELD score	22.67 ± 6.58	23.78 ± 8.91	
Blood type compatibility Yes No	30 0	29 1	
Cold ischemia time (hour)	6.04 ± 1.68	6.51 ± 2.45	
Warm ischemia time (min)	12.72 ± 6.88	13.34 ± 3.58	
Blood loss (mL/kg)	20.12 ± 9.45	21.85 ± 10.83	
Surgical duration (hour)	5.43 ± 1.01	6.03 ± 1.05	
Anhepatic phase (min)	45.73 ± 13.05	47.22 ± 9.08	

### 2.2 Characterization of bile exosomes

**FIGURE 1:**
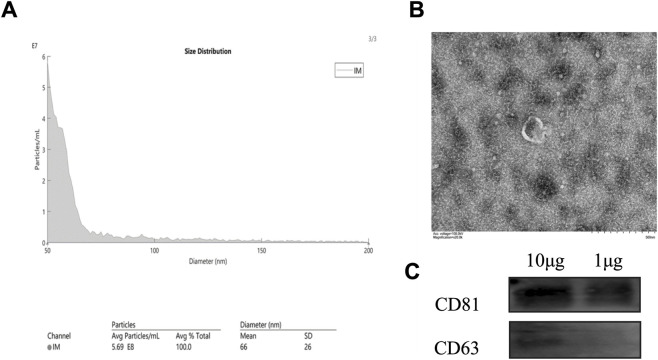
Characterization of bile exosomes from an AR recipient. **(A)** Measurement of bile exosome diameter from an AR recipient using nanoparticle tracking analysis. **(B)** Transmission electron micrograph of purified exosomes from the bile of an AR recipient. (scale bar: 500 nm). **(C)** Expression of exosome-specific marker proteins CD81 and CD63 detected by Western blotting.

**FIGURE 2:**
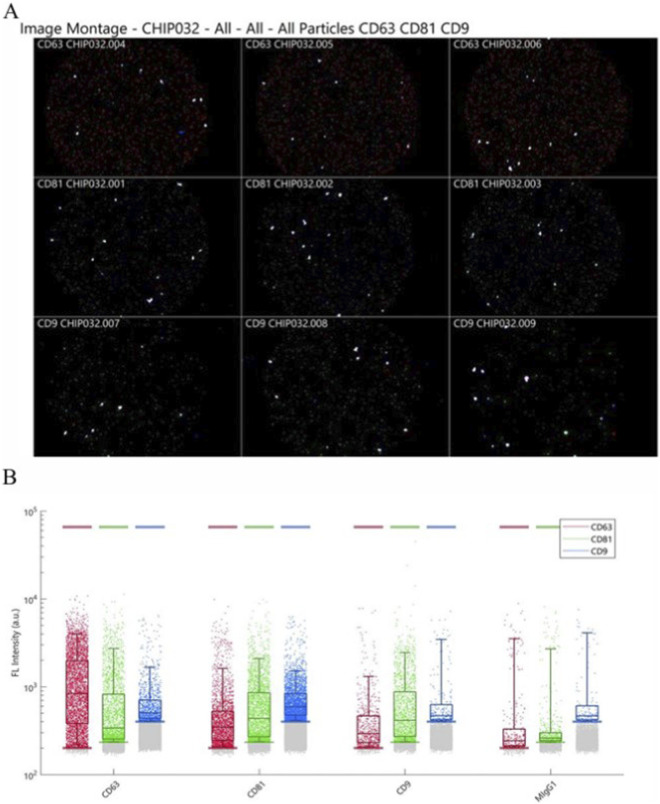
Characterization of exosome marker proteins in bile exosomes from an AR recipient. **(A)** Fluorescence images of the marker proteins CD63, CD81, and CD9 in bile exosomes from an AR recipient. **(B)** Fluorescence expression intensity of the marker proteins CD63, CD81, and CD9 in bile exosomes from an AR recipient.

In this study, exosomes were isolated from bile using ultracentrifugation. Nanoparticle tracking analysis characterized the particle size, showing an average diameter of approximately 66 nm, which is consistent with the typical size characteristics of exosomes (Figure 1A). Transmission electron microscopy revealed the typical cup-shaped morphology of the extracted biliary extracellular vesicles (Figure 1B). In addition, Western blotting detected the expression of CD81 and CD63, which are common key EV protein markers in exosomes (Figure 1C). Meanwhile, the NanoView system further confirmed the high expression of the exosomal marker proteins CD63, CD9, and CD81 (Figure 2). Collectively, these findings indicate that we have successfully obtained high-purity biliary exosomes suitable for subsequent molecular analyses.

### 2.3 High-throughput sequencing analysis of candidate differentially expressed miRNAs

**FIGURE 3:**
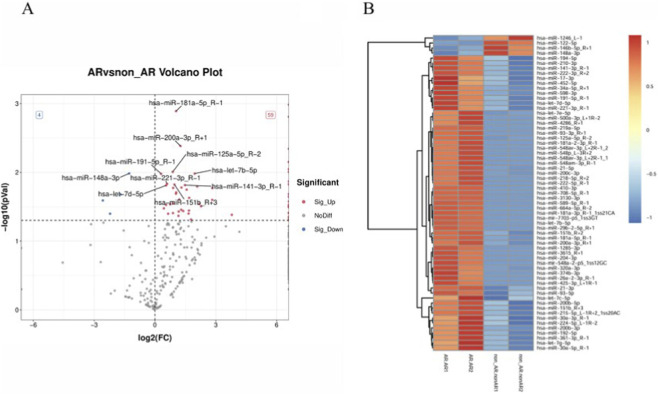
Volcano plot and heat map of candidate differentially expressed miRNAs in bile exosomes from AR and non-AR recipients. **(A)** Volcano plot. **(B)** Heat map.

High-throughput sequencing results showed that compared with non-AR recipients (n = 8), AR recipients (n = 8) had significantly elevated expression of 63 bile exosomal miRNAs and significantly decreased expression of 4 bile exosomal miRNAs. miRNAs with a |log2FC|>1 and consistent expression trends were defined as candidate differentially expressed miRNAs. Detailed results are presented in Figure 3, Supplementary Figure S1 and Table 2.

**TABLE 2:** Candidate differentially expressed miRNAs between groups (partial list) (AR vs. non-AR).

miRNA	Up/down	Fold change
hsa-miR-181a-5p_R-1	Up	2.05
hsa-miR-200a-3p_R+1	Up	2.39
hsa-miR-148a-3p	Down	0.41
hsa-let-7b-5p	Up	3.88
hsa-miR-141-3p_R-1	Up	2.78
hsa-miR-200c-3p	Up	4.22
hsa-miR-34a-5p_R+1	Up	7.11
hsa-miR-361-3p_R-1	Up	2.95
hsa-miR-151b_R+2	Up	2.06
hsa-miR-192-5p	Up	2.04
hsa-miR-122-5p	Down	0.31
hsa-miR-21-5p	Up	3.21
hsa-miR-223-5p_ R+1	Up	2.29
hsa-miR-210-3p	Up	7.08
hsa-miR-146b-5p_R+1	Down	0.17
hsa-miR-598-3p	Up	4.55
hsa-miR-21-3p	Up	4.84
hsa-miR-17-3p	Up	2.45
hsa-miR-181a-2-3p_R-1	Up	3.19
hsa-miR-200b-3p	Up	2.62
hsa-miR-93-5p	Up	2.25
hsa-miR-200b-5p	Up	3.15
hsa-miR-1246_L-1	Down	0.22
hsa-miR-452-5p	Up	13.85
hsa-let-7e-5p	Up	2.22
hsa-miR-500a-3p_L+1R-2	Up	3.43

### 2.4 Target gene function and pathway enrichment analysis of candidate differentially expressed miRNAs

**FIGURE 4:**
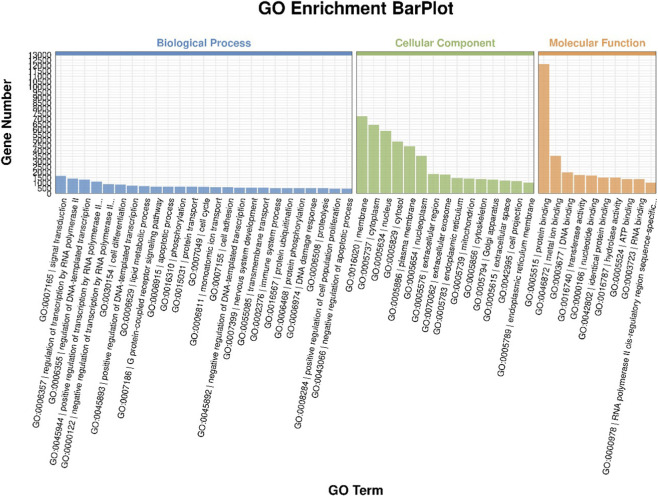
GO analysis of target genes of candidate differentially expressed bile exosomal miRNAs in AR recipients.

Target genes of the above miRNAs were predicted using RNAhybrid and miRanda software, followed by Gene ontology (GO) and Kyoto encyclopedia of genes and genomes (KEGG) enrichment analyses. The GO database annotates and analyzes gene functions from three aspects: cellular component, biological process, and molecular function. As shown in Figure 4, Supplementary Figure S2 and Supplementary Table S1, the candidate differentially expressed genes were largely involved in signal transduction, regulation of transcription by RNA polymerase II, negative regulation of transcription by RNA polymerase II, cell differentiation, positive regulation of DNA-templated transcription, lipid metabolic process, G protein-coupled receptor signaling pathway, apoptotic process, phosphorylation, protein transport, cell cycle, monoatomic ion transport, cell adhesion, negative regulation of DNA-templated transcription, nervous system development, transmembrane transport, immune system process, protein ubiquitination, protein phosphorylation, DNA damage response, proteolysis, positive regulation of cell population proliferation, and negative regulation of apoptotic process. These genes were enriched in membrane, cytoplasm, nucleus, cytosol, plasma membrane, nucleoplasm, extracellular region, extracellular exosome, endoplasmic reticulum, mitochondrion, cytoskeleton, Golgi apparatus, extracellular space, cell projection, and endoplasmic reticulum membrane. Genes related to molecular functions were primarily involved in protein binding, metal ion binding, DNA binding, transferase activity, nucleotide binding, identical protein binding, hydrolase activity, ATP binding, RNA binding, and RNA polymerase II cis-regulatory region sequence-specific DNA binding.

**FIGURE 5:**
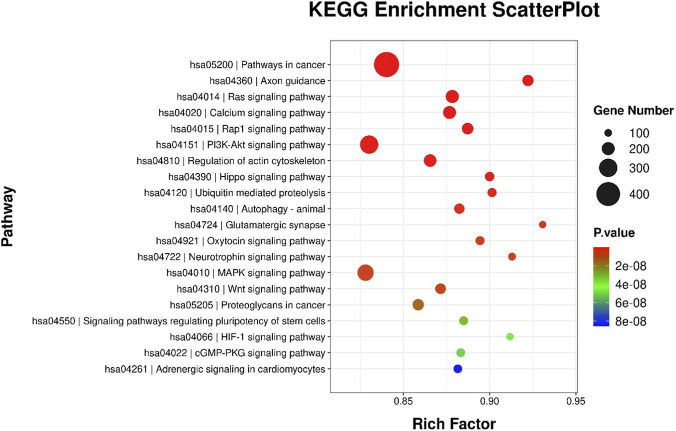
KEGG analysis of target genes of candidate differentially expressed bile exosomal miRNAs in AR recipients.

In AR recipients, the target genes of candidate differentially expressed bile exosomal miRNAs were significantly enriched in multiple KEGG pathways related to tumorigenesis, cell signal transduction, cytoskeletal regulation, autophagy, neural development, and others. Among these, pathways such as Pathways in cancer, Axon guidance, Ras signaling pathway, PI3K-Akt signaling pathway, MAPK signaling pathway, and Rap1 signaling pathway contained the largest numbers of target genes and showed strong enrichment with moderately high gene numbers, suggesting that these pathways may play key regulatory roles in the pathogenesis of AR. Furthermore, the enrichment of pathways including Hippo, Wnt, HIF-1, and cGMP-PKG further supports the involvement of exosomal miRNAs in transplant immune rejection through complex signaling networks (Figure 5; Supplementary Table S2).

### 2.5 Verification of miRNAs in bile exosomes from AR recipients

**FIGURE 6:**
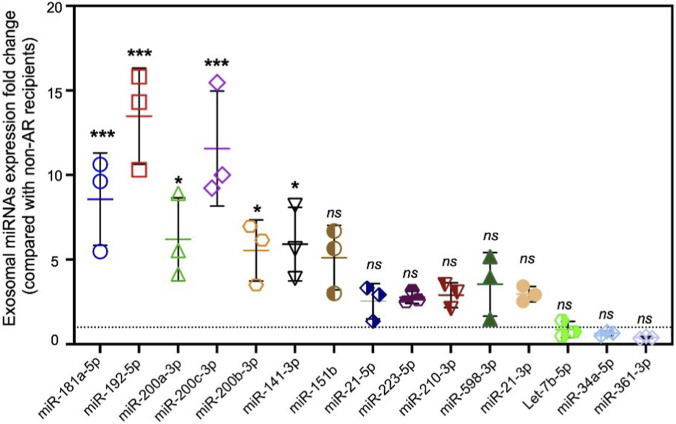
Differential expression of miRNAs in bile exosomes between AR and non-AR recipients.

The expression of miRNAs in bile exosomes from 22 AR to 22 non-AR recipients were detected by Real-Time Quantitative PCR (qRT-PCR). The data were analyzed using the 2^−ΔΔCt^ method and are presented in Figure 6. The results showed that the expression of miR-181a-5p, miR-200c-3p, and miR-192-5p were significantly higher in bile samples from AR than in those from non-AR recipients (P < 0.0001).

### 2.6 Correlation between bile exosomal miRNAs and clinical parameters

Spearman correlation analysis was performed to evaluate the associations between the expression levels of three bile exosomal miRNAs (miR-181a-5p, miR-200c-3p, and miR-192-5p) and key clinical parameters, including Banff rejection activity index (RAI), liver function biomarkers (alanine aminotransferase (ALT), alanine aminotransferase (AST), gamma glutamyl transferase (GGT), alkaline phosphatase (ALP), total bilirubin (TB)), and peripheral blood lymphocyte count (LYM). As shown in Table 3, miR-181a-5p expression exhibited a strong positive correlation with RAI (r = 0.712, P < 0.001), as well as with ALT (r = 0.654, P = 0.001) and AST (r = 0.623, P = 0.002). A moderate positive correlation was also observed between miR-181a-5p and TB (r = 0.512, P = 0.014). In contrast, miR-200c-3p showed the strongest correlation with GGT (r = 0.534, P = 0.009), followed by ALP (r = 0.487, P = 0.021) and TB (r = 0.456, P = 0.032), while its association with RAI score did not reach statistical significance (r = 0.398, P = 0.062). Notably, miR-192-5p was strongly correlated with ALT (r = 0.701, P < 0.001), AST (r = 0.678, P < 0.001), and RAI score (r = 0.623, P = 0.002), and also showed moderate correlations with TB (r = 0.589, P = 0.004) and GGT (r = 0.512, P = 0.014). None of the three miRNAs demonstrated significant correlations with LYM (P > 0.05 for all), suggesting that these bile exosomal miRNAs primarily reflect local intrahepatic immune injury rather than systemic immune status. Collectively, these findings indicate that bile exosomal miR-181a-5p and miR-192-5p are sensitive indicators of hepatocellular injury and rejection severity, whereas miR-200c-3p may be more closely associated with biliary injury.

**TABLE 3:** Correlations between bile exosomal miRNAs and clinical parameters.

Variable	miR-181a-5p	miR-200c-3p	miR-192-5p
RAI	**0.712**	NS	**0.623**
LYM(10^9^/L)	NS	NS	NS
ALT (U/L)	**0.654**	NS	**0.701**
AST (U/L)	**0.623**	NS	**0.678**
ALP (U/L)	NS	**0.487**	**0.423**
GGT (U/L)	NS	**0.534**	**0.512**
TB (μmol/L)	**0.512**	**0.456**	**0.589**

Values represent Pearson correlation coefficients (*r*). Only significant correlations (p < 0.05) are shown.

ALP, alkaline phosphatase; ALT, alanine aminotransferase; AST, aspartate aminotransferase; GGT, gamma glutamyl transferase; LYM, lymphocyte; NS, not significant; RAI, rejection activity index; TB, total bilirubin.

Bold values indicate statistically significant correlations (p < 0.05).

## 3 Discussion

AR after LT remains a major source of graft dysfunction and graft loss. In particular, acute cellular rejection is detected in up to 30% of recipients after LT, especially within the first year. To date, the exact pathogenesis of AR is not fully understood, and reliable non-invasive diagnostic biomarkers are still lacking (Krenzien et al., 2019; Pan et al., 2025). Furthermore, current diagnosis largely depends on invasive liver biopsy, and treatment options are limited to high-dose immunosuppression, which increases the risk of infection, malignancy, and other drug-related toxicities. Recurrent or refractory rejection may ultimately lead to graft failure and the need for retransplantation.

After LT, collecting bile *via* the intraoperatively placed T-tube is a clinically feasible and relatively non-invasive method for biological sampling (Pravisani et al., 2022). As a routine biliary drainage channel, the T-tube enables continuous and dynamic collection of native bile without additional punctures or endoscopic procedures, thereby avoiding the trauma and risks associated with repeated liver biopsy or ERCP. Bile is directly derived from the metabolic and secretory activities of hepatocytes and biliary epithelial cells and contains various components-such as bile acids, enzymes, cytokines, and miRNAs—that reflect liver function, ischemia-reperfusion injury, immune rejection, and biliary complications (Cox et al., 2024; Rauber et al., 2019; Umeshita et al., 1996; Buis et al., 2009). Bile exosomes are extracellular vesicles approximately 30–150 nm in diameter, released into bile *via* exocytosis by hepatocytes and biliary epithelial cells. These vesicles carry multiple active molecules from their parent cells, including proteins, lipids, and nucleic acids (e.g., miRNAs), and play a key role in intercellular communication.

As key regulators of gene expression, miRNAs are involved in immune-mediated diseases, including transplant rejection (Harris et al., 2010; Julian et al., 2025; Hamdorf et al., 2017). Bile, secreted by hepatocytes and modified by cholangiocytes, can early reflect pathological changes in the biliary epithelium and the graft immune microenvironment. Unlike blood, bile is directly secreted by the transplanted liver, offering a unique window for monitoring local immune responses. Bile exosomes are highly stable and locally enriched, acting as amplifiers to capture subclinical rejection signals undetectable in serum. Studies have shown that diagnostic miRNA marker combinations can be detected in biliary extracellular vesicles from patients with cholangiocarcinoma (Olaizola et al., 2018; Shu et al., 2024; Yoshida et al., 2023). Given that miRNAs within bile exosomes are protected by vesicles, exhibit strong stability, and can reflect the real-time pathophysiological state of the hepatobiliary system, bile exosomes hold significant clinical potential as biomarkers for the diagnosis and prognosis of hepatobiliary diseases (Yao et al., 2021), particularly biliary tract malignancies. Based on these advantages, focusing on bile exosomal miRNAs-shifting from systemic circulating markers to graft-localized exosome signatures-may provide a more specific and earlier non-invasive diagnostic strategy for AR after LT. This study revealed significant changes in the expression of specific miRNAs (such as miR-181a-5p, miR-200c-3p, and miR-192-5p) within bile exosomes from recipients with AR following LT.

miR-181a-5p is a conserved member of the miR-181 family and has been reported to participate in immune regulation, inflammatory responses, and cell signal transduction (Li et al., 2023). Previous studies have shown that miR-181a-5p can target genes in the Ras, MAPK, and PI3K-Akt signaling pathways (Lin et al., 2025; Ma et al., 2015), which are involved in T cell activation and inflammatory cytokine production (Mi et al., 2017; Zhang et al., 2024). Clinically, elevated miR-181a-5p expression has been observed in patients with acute rejection (Constanso-Conde et al., 2020; Millán et al., 2019; Millán et al., 2023; Wu et al., 2015). In line with these reports, our study found that miR-181a-5p was significantly upregulated in bile exosomes from AR recipients after LT. This finding extends previous serum-based observations to a more locally representative sample (bile exosomes), supporting the potential of miR-181a-5p as a non-invasive biomarker for AR monitoring. However, whether the upregulation of miR-181a-5p in bile exosomes directly reflects its regulatory roles in Ras/MAPK or PI3K-Akt pathways during AR remains to be experimentally determined.

The miR-200 family (miR-200a, miR-200b, miR-200c) has been implicated in inflammatory pathways and epithelial-mesenchymal transition (EMT) in various settings. In kidney transplantation, miR-200b is associated with interstitial fibrosis and tubular atrophy (Zu et al., 2017). Upregulation of miR-200c has been shown to inhibit the TLR4/MyD88/NF-κB pathway and reduce IL-6 and TNF-α levels, thereby alleviating acute rejection in experimental models (Wendlandt et al., 2012). In small intestine transplantation, exosomal miR-200b targets HMGB3 to suppress JNK activation and reduce inflammatory injury (Sun et al., 2020). These reports suggest that the miR-200 family may exert anti-inflammatory effects through TLR4/NF-κB and HMGB3/JNK pathways. In our study, we observed significant upregulation of miR-200 family members in bile exosomes during AR after LT. One speculative hypothesis is that this upregulation could represent a compensatory protective response of biliary epithelial cells under immune attack, possibly involving the PTEN/PI3K-Akt pathway and inhibition of NF-κB/TLR4 signaling, as suggested by *in silico* (Yang et al., 2019) target predictions. Nevertheless, this hypothesis is purely exploratory and requires direct experimental validation (e.g., using inflammatory cytokine-stimulated cholangiocytes *in vitro*). Regardless of the underlying mechanism, the elevated bile exosomal miR-200 levels highlight the value of bile as a liquid biopsy window for monitoring graft local microenvironment dynamics (Ottaviani et al., 2022).

miR-192 is highly expressed in liver tissue, predominantly in hepatocytes. Previous studies have reported elevated serum or plasma levels of miR-192 in hepatic ischemia-reperfusion injury (Roy et al., 2016), drug-induced liver injury (Bakshi et al., 2021), and after liver transplant rejection. Bij et al. (2017) analyzed 13 kidney transplant recipients with AR and found miR-192 markedly upregulated, with levels correlating with vascular injury markers (e.g., VEGF) and normalizing after successful anti-rejection therapy, suggesting that miR-192 may reflect microvascular endothelial injury and repair. In a rat LT model, (Hu et al., 2013) observed significantly upregulated plasma miR-192 during AR, which was suppressed by immunosuppressive therapy. In our study, the elevation of bile exosomal miR-192-5p may be related to hepatocyte injury and increased exosome secretion during rejection. Compared with serum, bile exosomal miR-192-5p originates directly from the transplanted liver and therefore may offer better organ specificity. Bioinformatic analyses have predicted a positive feedback loop between miR-192-5p and p53 (Moore et al., 2015), and inflammatory cytokines released during AR (e.g., IFN-γ, TNF-α) are known to induce p53 activation, which in turn can promote miR-192 transcription (Georges et al., 2008). However, whether such a feedback mechanism operates specifically in bile exosomes during acute rejection remains hypothetical and requires functional studies (e.g., luciferase reporter assays, miRNA gain/loss-of-function) to confirm.

Previous studies on biomarkers for AR after LT have mainly focused on circulating miRNAs in serum/plasma or miRNA expression profiles biopsy tissue (Yu and Lu, 2025; Wang et al., 2022). Although serum miRNA is relatively easy to obtain, its origin is complex and it is subject to interference from extrahepatic organs, immune cells, and systemic metabolic status, making it difficult to accurately reflect the local immune microenvironment of the graft. Tissue miRNA, while directly reflecting the molecular pathological status of the liver during rejection, requires invasive liver biopsy for acquisition, which carries risks of bleeding, bile leakage, sampling error, and poor patient compliance, thus limiting its use for dynamic monitoring. In contrast, this study reports for the first time a systematic identification and validation of the feasibility of bile exosomal miRNAs as diagnostic biomarkers for acute rejection, offering unique advantages and innovation. First, bile exosomal miRNAs more accurately reflect the “*in situ*” immune injury status of the graft, with less interference from other organs or systemic diseases, demonstrating organ specificity and local enrichment effects. Second, as carriers of intercellular communication, exosomes can actively encapsulate specific miRNAs, protecting them from degradation in the enzymatic environment of bile (containing RNases), thereby enabling stable detection. Meanwhile, the positive feedback regulatory loop identified in this study (mutual amplification between miR-192-5p and p53) may be specifically enriched in bile exosomes, generating a “signal amplification” feature that is more sensitive than that observed in serum or tissue. Bile obtained *via* an indwelling T-tube allows for non-invasive, repeatable sampling from the same patient, overcoming the bottleneck of frequent liver biopsy.

The pathways identified in this study are highly consistent with classical rejection mechanisms. The core mechanisms of classical AR include T cell activation and infiltration, release of inflammatory cytokines (IFN-γ, TNF-α), and apoptosis of target cells (hepatocytes/cholangiocytes). Several significantly altered miRNAs identified in this study fit into and link this process. Specifically, upregulation of miR-181 promotes T cell proliferation and effector function, thereby amplifying the immune attack. Inflammatory cytokines (IFN-γ, TNF-α) activate the p53 pathway in hepatocytes, and p53 transcriptionally upregulates miR-192-5p, forming a positive feedback amplification loop that exacerbates hepatocyte apoptosis. Meanwhile, altered expression of miR-200 may reflect cholangiocyte injury and repair imbalance. Collectively, these three miRNAs constitute a complete molecular chain from “immune recognition” to “target organ injury” to “structural remodeling,” providing a novel integrated perspective on the mechanisms of rejection.

The non-invasive diagnostic model based on bile exosomal miRNA signatures has significant clinical translational value. Bile can be continuously drained *via* a T-tube or obtained through minimally invasive methods during follow-up, enabling clinicians to detect changes in bile exosomal miRNA levels when liver function parameters (e.g., ALT, GGT, TBil) are still within borderline ranges or mildly elevated, thereby providing early warning of AR events days to weeks in advance. This allows for timely immunosuppressive intervention to prevent irreversible graft injury. Furthermore, this detection method is simple to perform and has a short turnaround time, making it suitable for dissemination in transplant centers with favorable health economic benefits.

This study also has several limitations. First, the sample size is relatively limited and derived from a single center, which may lead to overestimation of the diagnostic performance of the model. Its generalizability needs to be further validated by multicenter, large-scale, prospective studies. Second, the timing and frequency of bile collection have not been standardized, precluding precise determination of the temporal relationship between changes in bile exosomal miRNAs and the occurrence of rejection, and limiting the ability to detect subclinical or early mild rejection events. Third, the lack of standardization in exosome isolation methods and internal reference selection for miRNA detection compromises the comparability of results across different methods. Moreover, the current procedures are complex and equipment-intensive, hampering routine clinical implementation. Fourth, due to insufficient biological replicates, the sequencing lacked reliable differential expression power. Hence, all conclusions are drawn from qRT-PCR validation. Future studies should adopt individual sequencing (≥6 replicates/group) or pooled sequencing (≥3 replicates/group) for more reliable assessment. Fifth, although bioinformatic predictions and literature support functional mechanisms involving the miR-192-p53 positive feedback loop, miR-200 regulation of epithelial-mesenchymal transition, and miR-181 modulation of T cell activation, direct *in vitro* or *in vivo* functional validation experiments are lacking. Therefore, whether these miRNAs are directly pathogenic in rejection or merely act as “bystander” markers of injury remains inconclusive. Sixth, bile collection itself is still somewhat invasive (requiring a T-tube) and is not convenient for long-term survivors without an indwelling T-tube. Currently, it is more suitable for patients with an indwelling T-tube in the early postoperative period or as a screening tool prior to liver biopsy, rather than a complete replacement for liver biopsy. Seventh, the absence of exosomal negative marker evaluation precludes exclusion of potential contamination from endoplasmic reticulum or Golgi origin. Future validation of exosome purity will require inclusion of these markers. Furthermore, this study did not adequately include other types of graft injury as controls, such as ischemia-reperfusion injury, drug-induced liver injury, biliary obstruction, and viral infections, which may affect the diagnostic specificity of the model. Future studies should include these disease controls to further evaluate the clinical value of these miRNAs.

## 4 Materials and methods

### 4.1 Study subjects

From February 2022 to December 2025, bile samples were collected from liver transplant recipients *via* T-tubes. All recipients received organs from deceased citizens. Among them, 30 recipients were diagnosed with AR confirmed by biopsy, and the control group comprised 30 recipients with good recovery and stable liver function (non-AR) during the same period. Bile samples were collected within 48 h after AR diagnosis and before treatment, while for non-AR recipients, collection were matched to the corresponding postoperative course of the AR recipients. The screening cohort included 8 AR and 8 non-AR recipients, and the validation cohort included 22 AR and 22 non-AR recipients.

Inclusion criteria: ① Diagnosed with end-stage liver disease, excluding other infectious diseases, undergoing the first liver transplantation; ② Immunosuppressive medication regimen prescribed within 3 months, consisting of prednisone + mycophenolate mofetil + FK506 or cyclosporine; ③ Regular follow-up; ④ Signed informed consent form.

Exclusion criteria: ① Hyperacute or chronic rejection; ② Other complications such as bleeding, vascular stenosis, or vascular thrombosis within 1 month postoperatively; ③ Bacterial, tuberculosis, or fungal infections within 1 month postoperatively; ④ chronic wasting disease; ⑤ non-adherence to postoperative medication, examination, or follow-up.

This study was approved by the Ethics Committee of the First Affiliated Hospital of Xi’an Jiaotong University (No. 2022-201), and was conducted in accordance with the Declaration of Helsinki. All recipients provided written informed consent.

Collect bile samples, centrifuge at room temperature for 15 min at 2000 g to remove cells and debris, collect the supernatant, and store it in an ultra-low temperature freezer at −80 °C.

### 4.2 Exosome isolation

**FIGURE 7:**
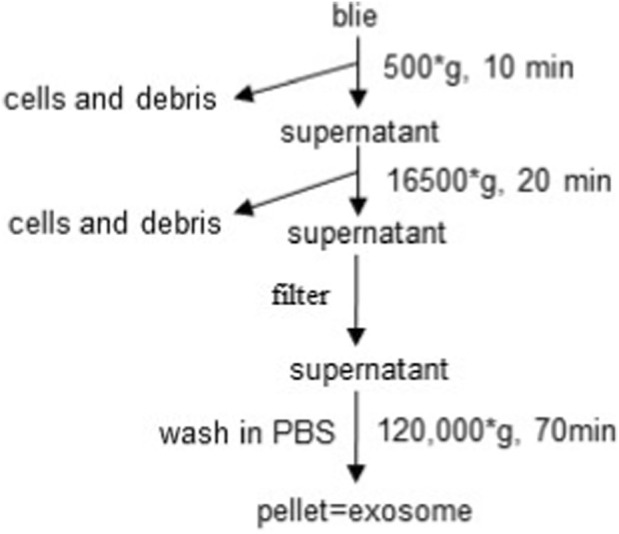
Flowchart of exosome isolation.

Thawed 10 mL of bile sample. After thawing, centrifuged at 500 *g* for 10 min at 4 °C to remove pelleted cells and debris, and collected the supernatant. Then centrifuged at 16,500 × g for 20 min at 4 °C to further remove cell debris, and filtered through a 200 nm filter. Transferred the supernatant to a centrifuge tube, washed with PBS, and centrifuged at 120,000 × g for 70 min at 4 °C. Yellow exosome pellets were obtained at the bottom of the centrifuge tube. The detailed step-by-step procedure was shown in Figure 7. The exosomes were used for immediate RNA sequencing, RNA extraction, or resuspended in PBS and stored at −80 °C.

### 4.3 Exosome identification

Transmission electron microscopy observation of exosomes was commissioned to Hangzhou Lianchuan Biotechnology Co., Ltd. Concentration and particle size distribution were analyzed using nanoparticle-tracking analysis. Exosome characterization was performed using the NanoView Automated Exosome Fluorescence Detection System. The sample was diluted with 1x sample dilution buffer to a concentration of 1 × 10^7^-1 × 10^8^ particles/mL. Then, 50 μL of the diluted sample was loaded onto a pre-scanned chip, sealed with parafilm, and incubated at room temperature for 16 h. The chip was washed three times (3 min each) with 1× Buffer under high-speed oscillation, followed by the addition of 250 μL of freshly prepared staining solution and incubation on a shaker in the dark for 1 h. Subsequently, the chip was washed three times (3 min each) with 1× Buffer B under high-speed oscillation, and then washed with deionized water under oscillation for 3 min. Using forceps to hold both sides of the chip, it was horizontally immersed in deionized water to remove surface impurities, then slowly lifted out of the water at a 45°angle to ensure no water droplets remained on the chip surface. The chip was air-dried on absorbent paper before detection. Data analysis for exosome characterization was performed based on capture antibodies immobilized on the chip, single-particle interferometric reflectance imaging sensor technology, and fluorescent signal labeling. Specific protein expression was analyzed using Western blotting for CD81 and CD63.

### 4.4 Exosome RNA extraction

Add 300 μL of lysis buffer A and 37.5 μL of lysis buffer B to 200 μL of resuspension containing purified exosomes. Mix thoroughly and incubate at room temperature for 10 min. Add 500 μL of 100% ethanol and vortex for 10 s. Transfer the mixture to a Mini Spin Column and centrifuge at 6,000 rpm for 1 min at room temperature. Repeat this step until all liquid has passed through the column. Add 600 μL of Elution Buffer A to the column and centrifuge at 6,000 rpm for 30 s at room temperature. Repeat this wash step once. Perform an empty spin at 14,000 rpm for 60 s at room temperature. Transfer the column to a new collection tube, add 50 μL of Elution Buffer, and centrifuge sequentially at 1,800 rpm for 60 s and then at 8,000 rpm for 120 s, both at room temperature. To maximize recovery, transfer the eluted buffer back onto the column, let stand at room temperature for 2 min, then centrifuge at 2,000 rpm for 1 min followed by 8,000 rpm for 2 min at room temperature.

### 4.5 Exosome RNA sequencing

RNA extracted from bile exosomes of 4 AR recipients was pooled into one tube for sequencing, and RNA from 4 non-AR recipients was pooled into one tube for sequencing. The obtained bile exosomal miRNAs were quality-checked using an Agilent 2100 Bioanalyzer, and the extracted RNA was then subjected to sequencing on an Illumina Hiseq 2000/2500.

### 4.6 Prediction of miRNA target genes and bioinformatics analysis

Targeted Scan (v5.0) and miRanda (v3.3a) were used to predict target genes, and their functions and potential biological pathways were completed through GO and KEGG databases.

### 4.7 qRT-PCR

RNA was reverse transcribed into cDNA using the RT first strand cDNA synthesis kit (Lot# LT203701). Quantitative expression analysis was performed on a PCR system using the qPCR SYBR Green Master Mix (No Rox) (CAT: 11201ES08), with each sample tested in triplicate. The 2^−ΔΔCt^ method was used to analyze the relative expression changes of miRNAs, with U6 serving as the internal control. The miRNA primer sequences were shown in Supplementary Table S3.

### 4.8 Statistical analysis

Statistical analysis was performed using GraphPad Prism, with results repeated three times. Quantitative data were expressed as mean ± standard deviation (X ± S). An independent samples t-test was used for comparisons between two groups. Spearman’s correlation analysis was performed to assess the correlation between bile exosomal miRNAs and clinical parameters. P value < 0.05 was considered statistically significant. Among them, *** indicates P < 0.001, ** indicates P < 0.01, and * indicates P < 0.05.

## 5 Conclusion

In conclusion, this study demonstrates that bile exosomal miRNAs can serve as potential candidate biomarkers for diagnosing AR after LT. Using a combination of small RNA sequencing and qRT-PCR validation, we identified and confirmed that miR-181a-5p, miR-200c-3p, and miR-192-5p are significantly upregulated in bile exosomes of AR recipients compared to those with non-AR recipients. Notably, the target genes of these candidate differentially expressed miRNAs are enriched in immune-related signaling pathways such as PI3K-Akt and MAPK, providing molecular insights into the underlying mechanisms of AR. These findings support the potential clinical utility of bile exosomal miRNA signatures as a low-risk, organ-specific liquid biopsy strategy for post-transplant monitoring. Further prospective studies with larger cohorts are warranted to validate their diagnostic accuracy and assess their role in guiding personalized immunosuppressive therapy.

## Data Availability

All data are available within this manuscript and its Supplementary Files. sRNA-seq data has been uploaded to SRA repository (PRJNA1464088).
