# Supplementary material for: Identification and validation of bile exosomal microRNA signatures for diagnosing acute rejection in liver transplant recipients
Source: Front Genet. 2026 Jun 29;17:1877181. doi: 10.3389/fgene.2026.1877181 (PMC13358000; doi:10.3389/fgene.2026.1877181)
Supplement: Supplementary file 1 [file DataSheet1.docx]

**Supplementary Information**

**Identification and Validation of Bile Exosomal MicroRNA Signatures for Diagnosing Acute Rejection in Liver Transplant Recipients**

Wenjing Wang^1^, Hui Wang^2^, Wen Li^2^, Renchi Fu^2^, Bo Wang^3^, Bo Guo^2*^

^1^ Surgical Intensive Care Unit, The First Affiliated Hospital of Xi’an Jiaotong University, Xi’an 710061, China; wangwenjing@xjtu.edu.cn (W.W.)

^2^ Department of Cell Biology and Genetics, School of Basic Medical Sciences, Xi’an Jiaotong University Health Science Center, Xi’an 710061, China; [wh092@stu.xjtu.edu.cn](mailto:wh092@stu.xjtu.edu.cn) (H.W.); liwen_61@xjtu.edu.cn (W.L.); [19891680534@stu.xjtu.edu.cn (R.F.)](mailto:19891680534@stu.xjtu.edu.cn%20(R.F.)); [bo_guo@xjtu.edu.cn](mailto:bo_guo@xjtu.edu.cn) (B.G.)

^3^ Department of Hepatobiliary Surgery, The First Affiliated Hospital of Xi’an Jiaotong University, Xi’an 710061, China; [bobwang75@sina.com](mailto:bobwang75@sina.com) (B.W.);

*** Correspondence:** [bo_guo@xjtu.edu.cn](mailto:bo_guo@xjtu.edu.cn)

**Table S1. Go enrichment analysis results (Parts of data)**

| GO_accession | Description | Term_type | p Value |
| --- | --- | --- | --- |
| GO:0005515 | protein binding | Molecular Function | 0 |
| GO:0005737 | cytoplasm | Cellular Component | 0 |
| GO:0005634 | nucleus | Cellular Component | 0 |
| GO:0005829 | cytosol | Cellular Component | 0 |
| GO:0005654 | nucleoplasm | Cellular Component | 3.0522E-223 |
| GO:0046872 | metal ion binding | Molecular Function | 1.4054E-211 |
| GO:0016740 | transferase activity | Molecular Function | 1.8346E-116 |
| GO:0016020 | membrane | Cellular Component | 3.8094E-108 |
| GO:0003677 | DNA binding | Molecular Function | 7.2778E-102 |
| GO:0000166 | nucleotide binding | Molecular Function | 6.32032E-98 |
| GO:0006357 | regulation of transcription by RNA polymerase II | Biological Process | 8.16702E-98 |
| GO:0005524 | ATP binding | Molecular Function | 6.79411E-78 |
| GO:0005794 | Golgi apparatus | Cellular Component | 1.09E-74 |
| GO:0000978 | RNA polymerase II cis-regulatory region sequence-specific DNA binding | Molecular Function | 8.57663E-74 |
| GO:0016787 | hydrolase activity | Molecular Function | 3.34644E-65 |
| GO:0042802 | identical protein binding | Molecular Function | 3.35836E-63 |
| GO:0005739 | mitochondrion | Cellular Component | 6.06149E-62 |
| GO:0000122 | negative regulation of transcription by RNA polymerase II | Biological Process | 3.50809E-61 |
| GO:0005783 | endoplasmic reticulum | Cellular Component | 1.37097E-60 |
| GO:0016310 | phosphorylation | Biological Process | 2.44092E-56 |
| GO:0015031 | protein transport | Biological Process | 5.04775E-56 |
| GO:0003723 | RNA binding | Molecular Function | 3.27341E-55 |
| GO:0045893 | positive regulation of DNA-templated transcription | Biological Process | 1.90217E-50 |
| GO:0070062 | extracellular exosome | Cellular Component | 3.1775E-49 |
| GO:0005789 | endoplasmic reticulum membrane | Cellular Component | 1.13135E-45 |
| GO:0006811 | monoatomic ion transport | Biological Process | 1.40989E-41 |
| GO:0030154 | cell differentiation | Biological Process | 3.78777E-41 |
| GO:0035556 | intracellular signal transduction | Biological Process | 2.28892E-37 |
| GO:0005886 | plasma membrane | Cellular Component | 1.16669E-32 |
| GO:0006915 | apoptotic process | Biological Process | 1.04253E-28 |
| GO:0006629 | lipid metabolic process | Biological Process | 1.07705E-27 |
| GO:0015629 | actin cytoskeleton | Cellular Component | 4.14236E-26 |
| GO:0007155 | cell adhesion | Biological Process | 1.19995E-25 |

**Table S2. KEGG pathway terms (Top 20)**

| pathway_term | rich_factor | p value | gene_number |
| --- | --- | --- | --- |
| Pathways in cancer | 0.8402 | 3.76105E-16 | 426 |
| Axon guidance | 0.9222 | 1.35074E-14 | 166 |
| Ras signaling pathway | 0.8783 | 5.4748E-12 | 202 |
| Calcium signaling pathway | 0.8767 | 1.17473E-11 | 199 |
| Rap1 signaling pathway | 0.8872 | 2.64874E-11 | 173 |
| PI3K-Akt signaling pathway | 0.8301 | 2.26309E-10 | 298 |
| Regulation of actin cytoskeleton | 0.8655 | 3.15325E-10 | 193 |
| Hippo signaling pathway | 0.9 | 4.03458E-10 | 135 |
| Ubiquitin mediated proteolysis | 0.9014 | 9.00287E-10 | 128 |
| Autophagy - animal | 0.8824 | 1.45347E-09 | 150 |
| Glutamatergic synapse | 0.9307 | 2.2947E-09 | 94 |
| Oxytocin signaling pathway | 0.8944 | 3.64624E-09 | 127 |
| Neurotrophin signaling pathway | 0.913 | 5.06984E-09 | 105 |
| MAPK signaling pathway | 0.828 | 5.37269E-09 | 260 |
| Wnt signaling pathway | 0.8715 | 5.42679E-09 | 156 |
| Proteoglycans in cancer | 0.8586 | 1.36681E-08 | 170 |
| Signaling pathways regulating pluripotency of stem cells | 0.8849 | 3.02391E-08 | 123 |
| HIF-1 signaling pathway | 0.9118 | 4.72026E-08 | 93 |
| cGMP-PKG signaling pathway | 0.8832 | 5.0922E-08 | 121 |
| Adrenergic signaling in cardiomyocytes | 0.8815 | 8.5427E-08 | 119 |

**Table S3.** List of miRNA primers

| miRNA | Sequence (5’-3’) |
| --- | --- |
| miRNA-F | ATCCAGTGCGTGTCGTG |
| miRNA-181a-5p-RT | GTCGTATCCAGTGCGTGTCGTGGAGTCGGCAATTGCACTGGATACGACACTCACC |
| miRNA-181a-5p-R | TGCTAACATTCAACGCTGTC |
| miRNA-192-5p-RT | GTCGTATCCAGTGCGTGTCGTGGAGTCGGCAATTGCACTGGATACGACGGCTGTC |
| miRNA-192-5p-R | TGCTCTGACCTATGAATT |
| miRNA-200a-3p-RT | GTCGTATCCAGTGCGTGTCGTGGAGTCGGCAATTGCACTGGATACGACACATCGT |
| miRNA-200a-3p-R | TGCTTAACACTGTCTGGTA |
| miRNA-200c-3p-RT | GTCGTATCCAGTGCGTGTCGTGGAGTCGGCAATTGCACTGGATACGACTCCATCA |
| miRNA-200c-3p-R | TGCTTAATACTGCCGGGTAA |
| miRNA-200b-3p-RT | GTCGTATCCAGTGCGTGTCGTGGAGTCGGCAATTGCACTGGATACGACTCATCAT |
| miRNA-200b-3p-R | TGCTTAATACTGCCTGGTA |
| miRNA-141-3p-RT | GTCGTATCCAGTGCGTGTCGTGGAGTCGGCAATTGCACTGGATACGACCCATCTT |
| miRNA-141-3p-R | TGCTTAACACTGTCTGGTA |
| miRNA-151b-RT | GTCGTATCCAGTGCGTGTCGTGGAGTCGGCAATTGCACTGGATACGACAGACTGT |
| miRNA-151b-R | TGCTTCGAGGAGCTC |
| miRNA-21-5p-RT | GTCGTATCCAGTGCGTGTCGTGGAGTCGGCAATTGCACTGGATACGACTCAACAT |
| miRNA-21-5p-R | TGCTTAGCTTATCAGACTG |
| miRNA-223-5p-RT | GTCGTATCCAGTGCGTGTCGTGGAGTCGGCAATTGCACTGGATACGACAACTCAG |
| miRNA-223-5p-R | TGCTCGTGTATTTGACAAG |
| miRNA-210-3p-RT | GTCGTATCCAGTGCGTGTCGTGGAGTCGGCAATTGCACTGGATACGACTCAGCCG |
| miRNA-210-3p-R | TGCTCTGTGCGTGTGACAG |
| miRNA-598-3p-RT | GTCGTATCCAGTGCGTGTCGTGGAGTCGGCAATTGCACTGGATACGACTGACGAT |
| miRNA-598-3p-R | TGCTTACGTCATCGTTGTC |
| miRNA-21-3p-RT | GTCGTATCCAGTGCGTGTCGTGGAGTCGGCAATTGCACTGGATACGACACAGCCC |

| miRNA | Sequence (5’-3’) |
| --- | --- |
| miRNA-21-3p-R | TGCTCAACACCAGTCGAT |
| let-7b-5p-RT | GTCGTATCCAGTGCGTGTCGTGGAGTCGGCAATTGCACTGGATACGACAACCACA |
| let-7b-5p-R | TGCTTGAGGTAGTAGGTTG |
| miRNA-34a-5p-RT | GTCGTATCCAGTGCGTGTCGTGGAGTCGGCAATTGCACTGGATACGACACAACCA |
| miRNA-34a-5p-R | TGCTTGGCAGTGTCTTAGC |
| miRNA-361-3p-RT | GTCGTATCCAGTGCGTGTCGTGGAGTCGGCAATTGCACTGGATACGACAAATCAG |
| miRNA-361-3p-R | TGCTTCCCCCAGGTGTGATT |
| U6-RT | CGCTTCACGAATTTGCGTGTCAT |
| U6-F | GCTTCGGCAGCACATATACTAAAAT |
| U6-R | CGCTTCACGAATTTGCGTGTCAT |


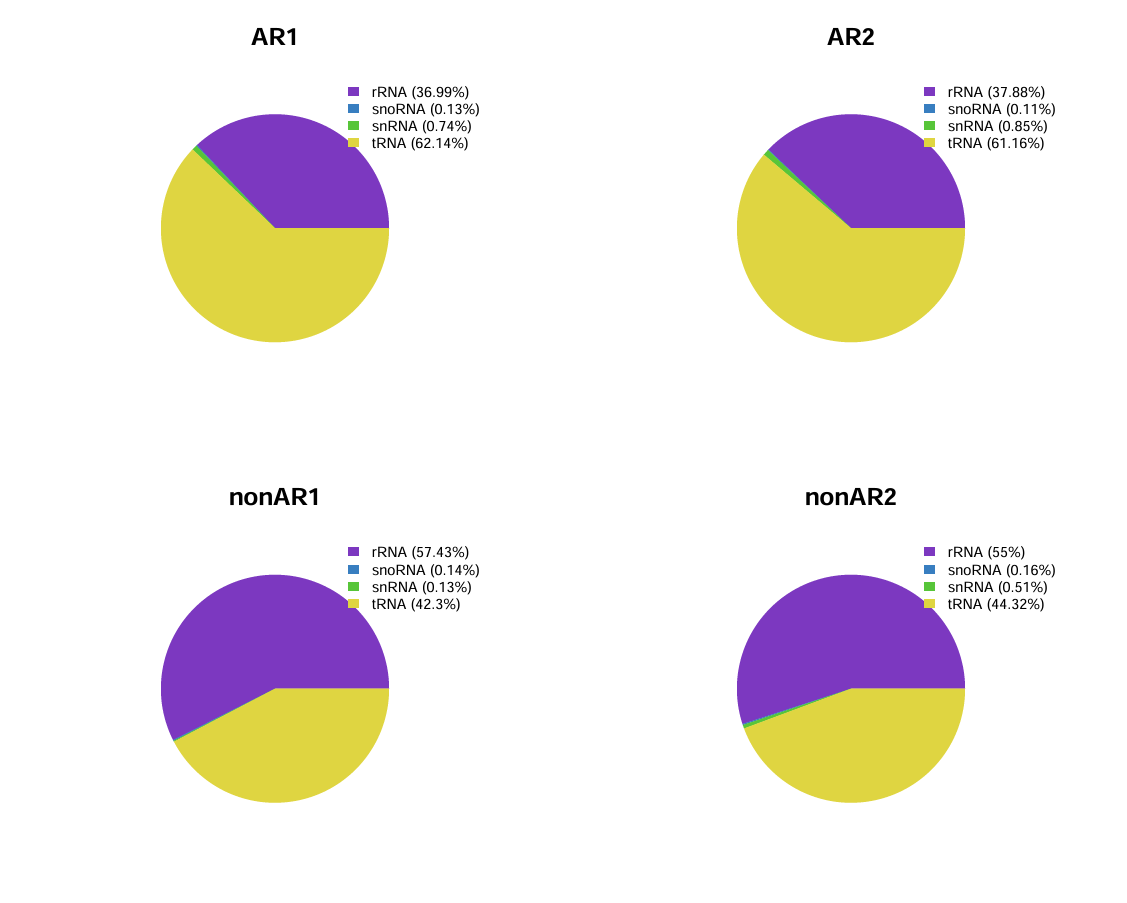


**Figure S1**. The ncRNA classification annotation statistics of the clean reads

**
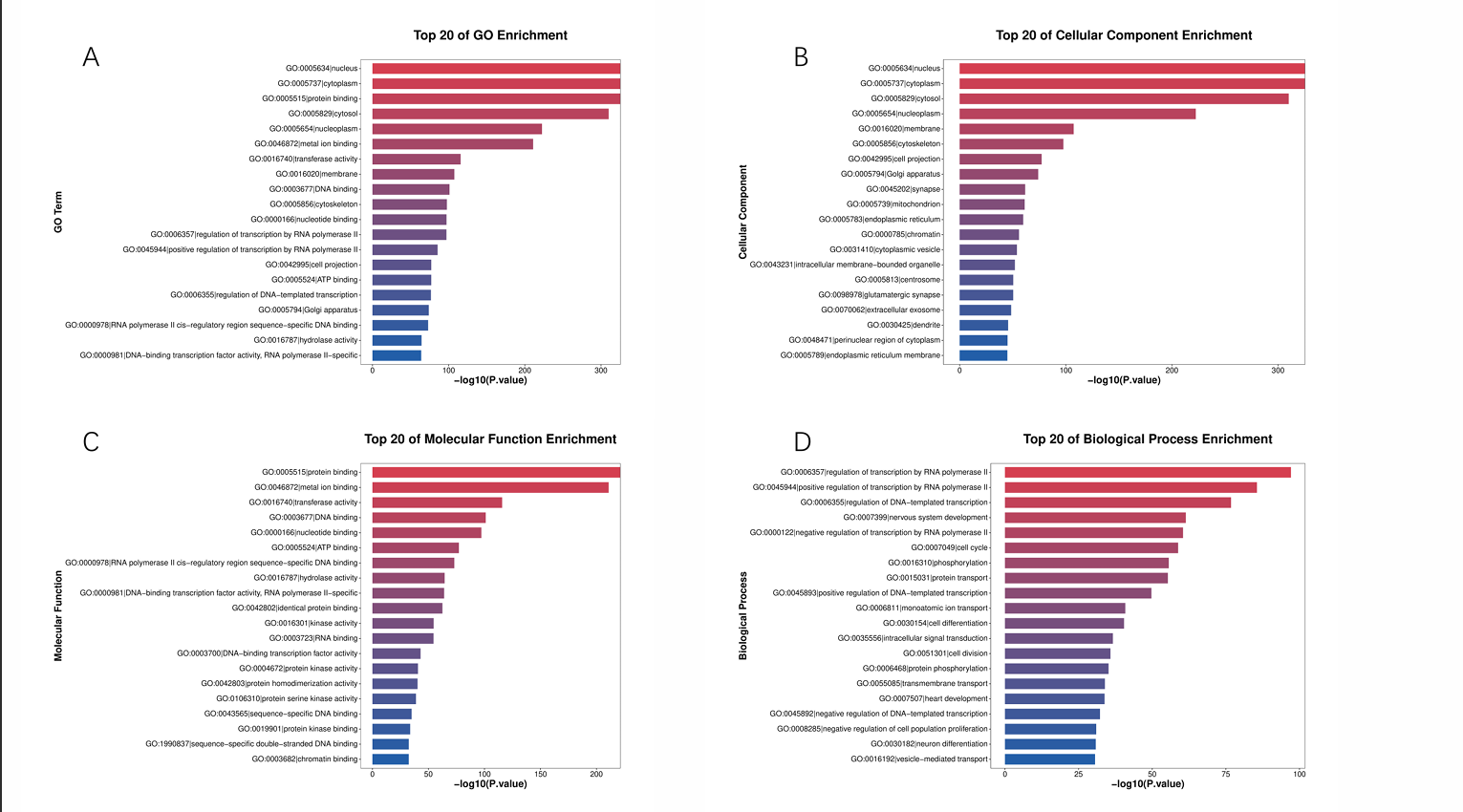
**

**Figure S2**. GO function classification target genes of known differential expressed miRNAs in AR recipients. **(A)** Top 20 of GO enrichment. **(B)** Top 20 of cellular component enrichment. (C) Top 20 of molecular function enrichment. **(D)** Top 20 of biological process enrichment.
